# Twinkle artifact in sonographic breast clip visualization

**DOI:** 10.1007/s00404-022-06659-1

**Published:** 2022-07-13

**Authors:** Banys-Paluchowski Maggie, Paluchowski Peter, Krawczyk Natalia

**Affiliations:** 1 Department of Gynecology and Obstetrics, University Hospital Schleswig-Holstein Campus Lübeck, Lübeck, Germany; 2 Department of Gynecology and Obstetrics, Breast Cancer Center, Regio Klinikum Pinneberg, Pinneberg, Germany; 3 Department of Gynecology and Obstetrics, Heinrich Heine University Düsseldorf, Düsseldorf, Germany

**Keywords:** Twinkle artifact, Twinkling artifact, Breast ultrasound, Clip, Targeted axillary dissection

## Abstract

The ‘twinkle’ or ‘twinkling’ artifact represents a phenomenon observed using color Doppler ultrasound that leads to a rapid alternation of color in and immediately behind an echogenic and highly reflective object. It occurs during sonographic examination of kidney stones, and has been also described in clips used for marking breast and axillary lesions.

**Supplementary Information:**

The online version contains supplementary material available at 10.1007/s00404-022-06659-1.

While the exact nature of this artifact remains poorly understood, it may prove useful when localizing a clip, for example for a targeted axillary dissection (TAD) in breast cancer patients receiving neoadjuvant chemotherapy (NACT). This surgical technique consists of the removal of a target lymph node, i.e., a biopsy-proven and marked node, and sentinel node biopsy, and can be offered patients converting from positive to negative node status through NACT (cN +  → ycN0). Usually, the target node is marked using a clip/coil, but other probe-guided detection techniques, such as magnetic or radar localization, may be used as well [1]. In case a clip has been placed into the node, its detection depends mainly on its reliable ultrasound visibility. For this reason, larger clips and those with a 3D shape or hydrogel carrier are often chosen, but the ultrasound detection rate remains lower than expected (approx. 70–90% in previous studies). Therefore, additional tools such as the twinkle artifact may help to identify the clip. However, not all clip types produce this artifact, so the documentation of exact clip type and shape is recommended (Fig. 1).

**Fig. 1:**
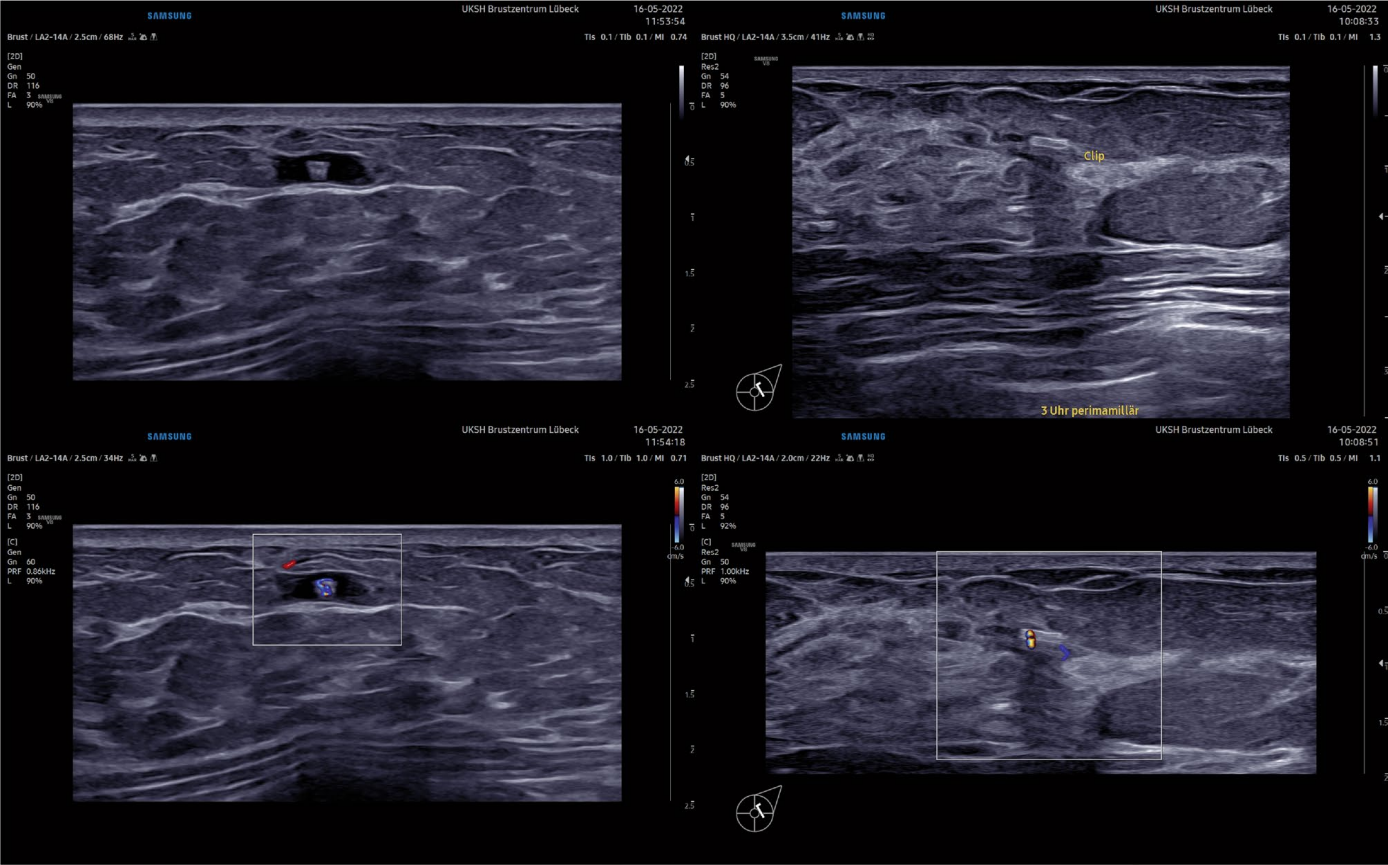
Two different clips shown in B-mode (upper row) and using color Doppler (lower row). Left: small cavernous hemangioma, confirmed by core-biopsy with synchronous clip placement. The visible lesion with the clip was localized intraoperatively via ultrasound without the necessity for a preoperative localization step with, e.g., a wire. Right: core-biopsy confirmed breast cancer with complete remission upon imaging. The clip was placed before start of neoadjuvant chemotherapy

## Supplementary Information

Below is the link to the electronic supplementary material.

Supplementary file1 (AVI 1557 KB)

Supplementary file2 (AVI 11184 KB)
